# Clinical utility of subgingival plaque-specific bacteria in salivary microbiota for detecting periodontitis

**DOI:** 10.1371/journal.pone.0253502

**Published:** 2021-06-25

**Authors:** Jiale Ma, Shinya Kageyama, Toru Takeshita, Yukie Shibata, Michiko Furuta, Mikari Asakawa, Yoshihisa Yamashita

**Affiliations:** 1 Section of Preventive and Public Health Dentistry, Division of Oral Health, Growth and Development, Faculty of Dental Science, Kyushu University, Fukuoka, Japan; 2 OBT Research Center, Faculty of Dental Science, Kyushu University, Fukuoka, Japan; New York Medical College, UNITED STATES

## Abstract

Saliva contains diverse bacteria shed from various oral sites, including subgingival plaque. It is reasonable to focus on the total occupancy of subgingival plaque-specific bacteria (SUBP bacteria), which live in subgingival environments, in the saliva for detecting periodontitis using salivary testing. This study aimed to validate the clinical utility of SUBP bacteria in the salivary microbiota for the detection of periodontitis. We examined stimulated saliva samples collected from 125 subjects who visited three dental clinics. The relative abundances of previously identified 11 SUBP bacteria were determined using 16S ribosomal RNA gene sequencing and a reference-based approach. The prediction performance was evaluated using a receiver operating characteristic (ROC) curve. The SUBP bacteria accounted for 0–15.4% of the salivary microbiota, and the percentage distinguished periodontitis patients with at least 15 sites with probing depth ≥4 mm with a sensitivity of 0.90 (95% confidence interval [CI], 0.81–0.98) and specificity of 0.70 (95% CI, 0.60–0.80) (area under the ROC curve [AUC], 0.87). Among 2,047 combinations of 11 SUBP bacteria, combinations including *Streptococcus constellatus*, *Porphyromonas gingivalis*, and *Fusobacterium nucleatum* subsp. *vincentii* demonstrated significantly higher AUC values in their detection. These results suggest that examining SUBP bacteria in saliva may be useful for detecting periodontitis patients in mass screening.

## Introduction

Periodontitis is an infectious disease consequent to a complex interaction between host immune response and dental plaque microorganisms and is clinically characterized by alveolar bone resorption and deep periodontal pocket formation. The subgingival space of the periodontal pocket provides an anaerobic habitat to oral microorganisms and is occupied by obligatory anaerobic and proteolytic subgingival bacteria. For instance, *Porphyromonas gingivalis*, *Tannerella forsythia*, and *Treponema denticola* are classically well-known as subgingival bacteria and called "red complex" because of their co-aggregation characteristics and strong association with diseased sites [1, 2]. In a recent systematic review comparing studies using culture-independent microbiological approaches, several bacterial species, which showed higher levels of proportion, prevalence, or abundance in subgingival plaque of periodontitis patients, were found to be novel subgingival bacteria [3]. These subgingival bacteria construct a complex and unique microbial ecosystem in the subgingival plaque of periodontitis patients. Therefore, a large number of studies using various approaches have examined subgingival bacteria in order to reveal the etiology of periodontitis [4–8].

Although subgingival plaque samples are secure specimens for examining the subgingival microbiota, saliva samples have recently been considered alternative specimens to assess the overall subgingival microbiota because saliva contains microorganisms shed from various oral niches, including subgingival plaque. Several studies have reported a strong correlation between the microbial profile of pooled subgingival plaque and saliva samples [9–13]. Considering that inflammation of periodontal tissue broadly spreads with alveolar bone resorption and deepening of the periodontal pocket, it is reasonable that the proportion of subgingival bacteria in the salivary microbiota commensurately increased with the progression of periodontitis. In our previous study, we comprehensively identified 12 subgingival plaque-specific bacteria (SUBP bacteria), which specifically inhabit subgingival plaque, by examining various oral samples (supra- and subgingival plaque, tongue coating, and saliva samples) collected from patients with severe periodontitis, and found that the total relative abundance of the 12 SUBP bacteria in the salivary microbiota was correlated with the percentage of sites with probing depth ≥4 mm [12]. Interestingly, these SUBP bacteria were composed of not only known pathogens but also bacterial species whose pathogenicity to periodontitis was unclear, and the total relative abundance in saliva more strongly reflected periodontal status than that in each SUBP bacteria alone. Although some previous studies focused on well-known periodontal pathogens in saliva to detect periodontitis patients [14, 15], focusing on the total bacterial occupancy in saliva of these SUBP bacteria that live in subgingival environments regardless of virulence seems to be innovative and more reasonable for detecting periodontitis in salivary testing. However, the prediction performance of SUBP bacteria in saliva has not yet been validated using large populations with a wide range of severity.

In this study, we examined the salivary microbiota of subjects who visited three dental offices using 16S ribosomal RNA (rRNA) gene sequencing. In particular, we focused on only 12 previously identified SUBP bacteria and calculated the total relative abundance of these bacteria in salivary microbiota using a reference-based approach. The purpose of this study was to validate the clinical utility of SUBP bacteria in salivary microbiota for the detection of periodontitis using a larger sample size with a wide range of severity.

## Materials and methods

### Study subjects and sample collection

The subjects of this study were 129 individuals who visited three dental clinics in three different prefectures: Saitama, Tottori, and Fukuoka. Of the 129 subjects, 14 were previously studied, and 115 were additionally enrolled in this study [12]. Subjects who used antibiotics within a month preceding sampling were not recruited. Dental examinations and saliva sample collection were conducted following a previously described protocol [16]. We instructed the subjects to chew gum for 5.5 min and to discharge the whole saliva into sterile plastic tubes during the final 5 min. The probing depth (PD) at six sites (mesiobuccal, midbuccal, distobuccal, mesiolingual, midlingual, and distolingual) in all teeth was measured using a periodontal pocket probe after sample collection. The number of remaining teeth and total probing sites were also recorded during the clinical evaluation. After excluding four subjects who had missing clinical data (n = 3) or whose saliva samples were insufficient for analysis (n = 1), 125 subjects were finally included in this study. Written informed consent was obtained from all participants. The ethics committee of Kyushu University approved this study and the informed consent procedure (approval number 2019–105).

### 16S rRNA gene amplicon sequencing of saliva

DNA was extracted from each newly collected sample using the bead-beating method [16], and the V1–V2 regions of the 16S rRNA gene were amplified using the following primers: 8F (5-AGA GTT TGA TYM TGG CTC AG-3) with the Ion Torrent adapter A and the sample-specific 8-base tag sequence and 338R (5-TGC TGC CTC CCG TAG GAG T-3) with the Ion Torrent trP1 adapter sequence. PCR amplification, purification, and quantification of each PCR amplicon was performed as previously described [17]. The purified PCR amplicons were pooled, and gel purification was performed using the Wizard SV Gel and PCR Clean-Up System (Promega, WI, USA). The DNA concentration was determined using a KAPA Library Quantification Kit (KAPA Biosystems, MA, USA), and the DNA was diluted for use as the template DNA in emulsion PCR. Emulsion PCR and enrichment of template-positive particles were performed using Ion PGM Template Hi-Q View OT2 Kit (Thermo Fisher Scientific) in Ion One Touch 2 system (Thermo Fisher Scientific). The enriched particle was loaded onto Ion 318 v2 chips (Thermo Fisher Scientific), and sequencing was performed on the Ion PGM (Thermo Fisher Scientific) using the Ion PGM Hi-Q view Sequencing Kit (Thermo Fisher Scientific).

### Data analysis and taxonomy assignment

Quality filtering of all raw sequence reads was performed using a script written in R (version 3.6.2). The reads were excluded from the analysis when they exhibited ≤200 bases, had an average quality score ≤25, did not include the correct forward primer sequence, the correct reverse primer sequence (one mismatch was allowed), or had a homopolymer of >6 nucleotides. The quality-checked reads were demultiplexed by examining the 8-base tag sequence, and the forward and reverse primer sequences were trimmed. The taxonomy of each quality-checked read was directly determined using BLAST against 889 oral bacterial 16S rRNA gene sequences (16S rRNA RefSeq version 14.51) in the human oral microbiome database (HOMD) with 98.5% identity [18, 19]. In this study, we focused on 12 bacterial species, previously identified as SUBP bacteria [12]. Of the 12 SUBP bacteria, the total relative abundances of only 11 SUBP bacteria, except one previously not assigned at the species level, were calculated based on each hit and total read number. The sequence data have been deposited in the DDBJ sequence read archive (DRA005104 and DRA011902).

### Statistical analysis

In this study, we defined six criteria for periodontal status based on the number of sites with PD ≥4 mm (≥1, ≥3, ≥5, ≥10, ≥15, and ≥30 sites). The drawing of receiver operating characteristic (ROC) curves, calculation of area under the curve (AUC) values, computation of confidence intervals (CIs) of AUCs, sensitivity, and specificity, and estimation of sample size were performed using the pROC package in R [20]. With an expected AUC value of 0.80 and a total of 125 subjects (0.19–0.80 of case proportion), it was estimated that the statistical power for this study reached 0.99 for all criteria. Optimal cutoff values were determined based on the Youden index [21]. Finally, we explored bacterial species that particularly affect the prediction of periodontal status among the 11 SUBP bacteria. We computed the total relative abundances of 2,047 combinations of 11 SUBP bacteria (_11_C_1_, _11_C_2_… _11_C_11_) and calculated each AUC value. The AUC values of combinations with and without SUBP bacteria were compared using the Mann-Whitney U test, and P-values were adjusted using a Benjamini-Hochberg false discovery rate (FDR) correction for multiple testing. All statistical analyses were performed using the R software (version 3.6.2).

## Results

### The characteristics of subjects and salivary microbiota sequence

We examined 125 subjects (47 men and 78 women, aged 22–91 years) who visited three dental offices. The detailed characteristics of the study subjects are presented in Table 1. Of the 125 subjects, 20% were periodontally healthy with no site with PD ≥4 mm and 80% had at least one site with PD ≥4 mm. Regarding the distribution of subjects, subjects with ≥30 sites with PD ≥4 mm were most commonly found (19.2%), followed by subjects with 5–9 sites with PD ≥4 mm (16.8%). We analyzed their stimulated saliva samples by 16S rRNA gene amplicon analysis, and finally obtained 2,228,858 high-quality reads (17,830.9 ± 5,022.4 reads per sample) to determine the relative abundances of 11 SUBP bacteria.

**Table 1:** The clinical characteristics of study subjects.

Characteristic	Subjects (n = 125)
Age (years), mean ± SD	55.4 ± 16.2
Gender, n (%)	
Male	47 (37.6)
Female	78 (62.4)
Number of teeth, mean ± SD	25.8 ± 4.0
Number of sites with probing depth ≥4 mm, n (%)	
None	25 (20.0)
1–2 sites	18 (14.4)
3–4 sites	13 (10.4)
5–9 sites	21 (16.8)
10–14 sites	6 (4.8)
15–29 sites	18 (14.4)
≥30 sites	24 (19.2)

SD, standard deviation

### SUBP bacteria in salivary microbiota

We calculated the relative abundances of each SUBP bacteria and the total relative abundance of 11 SUBP in the salivary microbiota (Table 2). Although SUBP bacteria were minor components of the salivary microbiota, all SUBP bacteria were observed. Among the 11 SUBP bacteria, *Fusobacterium nucleatum* subsp. *vincentii* was the most broadly abundant (median of relative abundance: 0.032%), and *Porphyromonas gingivalis* demonstrated the highest relative abundance (maximum relative abundance: 6.44%). In total, SUBP bacteria accounted for 0–15.4% of their salivary microbiota.

**Table 2:** Relative abundances of the SUBP bacteria in salivary microbiota.

SUBP bacteria	Relative abundance (%), Median (range)
*Fusobacterium nucleatum* subsp. *vincentii* HOT200	0.032 (0–1.86)
*Parvimonas micra* HOT111	0.013 (0–2.30)
*Streptococcus constellatus* HOT576	0.009 (0–0.73)
*Porphyromonas gingivalis* HOT619	0.008 (0–6.44)
*Tannerella forsythia* HOT613	0.006 (0–1.47)
*Porphyromonas endodontalis* HOT273	0.005 (0–1.54)
*Fusobacterium nucleatum* subsp. *nucleatum* HOT698	0 (0–2.42)
*Filifactor alocis* HOT539	0 (0–1.07)
*Fretibacterium* sp. HOT359	0 (0–0.12)
*Desulfobulbus* sp. HOT041	0 (0–0.11)
*Fusobacterium* sp. HOT370	0 (0–0.05)
Total of 11 SUBP bacteria	0.14 (0–15.38)

Human oral taxon (HOT) numbers in the human oral microbiome database (HOMD) are given following bacterial names.

### Prediction of periodontal status using SUBP bacteria in salivary microbiota

**Fig 1:**
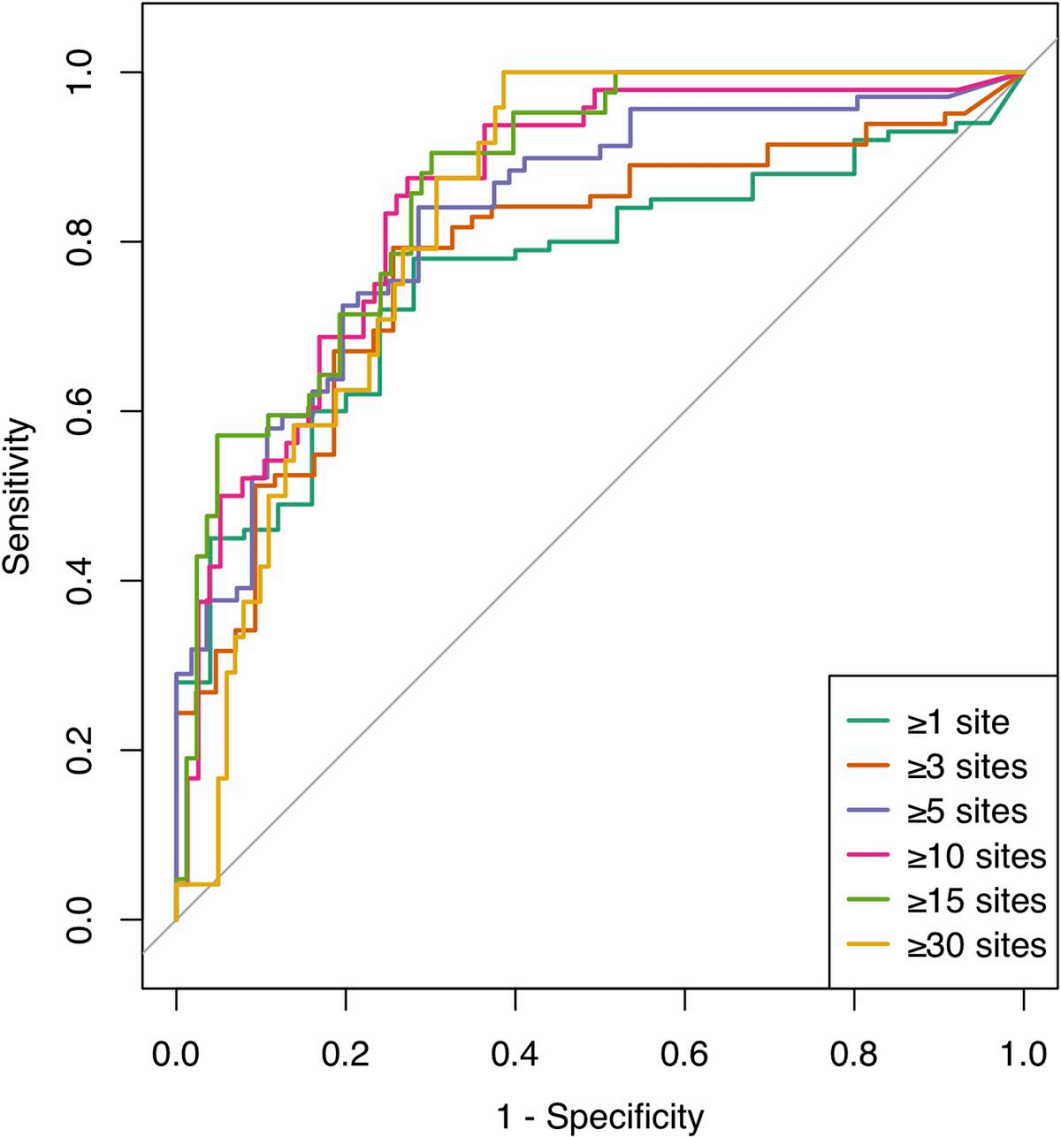
Receiver operating characteristic (ROC) curves for prediction of periodontal status using the total relative abundance of the SUBP bacteria in salivary microbiota. Periodontal status was defined by the six criteria (≥1, ≥3, ≥5, ≥10, ≥15, and ≥30 sites with PD ≥4 mm), and depicted using different colors.

We validated whether SUBP bacteria in the salivary microbiota could detect periodontitis patients with various severities. Fig 1 shows the ROC curves for the prediction of periodontal status based on the six criteria using the total relative abundance of 11 SUBP bacteria. The prediction performance of each criterion is presented in Table 3. Among the six criteria, the highest AUC value was obtained in the criterion of periodontal status with ≥15 sites with PD ≥4 mm (AUC, 0.87; 95% CI, 0.81–0.93). When we set the cutoff of the total relative abundance of SUBP bacteria to 0.139, we identified the subjects with this periodontal status with a sensitivity of 0.90 (95% CI, 0.81–0.98) and specificity of 0.70 (95% CI, 0.60–0.80).

**Table 3:** Prediction performance of the total relative abundance of the SUBP bacteria.

Periodontal status	AUC (95% CI)	Cutoff (%)	Sensitivity (95% CI)	Specificity (95% CI)
≥1 site with PD ≥4 mm	0.76 (0.67–0.85)	0.057	0.78 (0.70–0.86)	0.72 (0.56–0.88)
≥3 sites with PD ≥4 mm	0.78 (0.70–0.87)	0.074	0.79 (0.71–0.88)	0.74 (0.6–0.86)
≥5 sites with PD ≥4 mm	0.83 (0.76–0.90)	0.079	0.84 (0.75–0.93)	0.71 (0.59–0.84)
≥10 sites with PD ≥4 mm	0.85 (0.78–0.92)	0.139	0.88 (0.77–0.96)	0.73 (0.62–0.82)
≥15 sites with PD ≥4 mm	0.87 (0.81–0.93)	0.139	0.90 (0.81–0.98)	0.70 (0.60–0.80)
≥30 sites with PD ≥4 mm	0.83 (0.76–0.91)	0.139	1.00 (1.00–1.00)	0.61 (0.52–0.70)

PD, probing depth; CI, confidence interval; AUC, the area under the receiver operating characteristics (ROC) curve.

### Exploring of impactful SUBP bacteria on prediction of periodontal status

**Fig 2:**
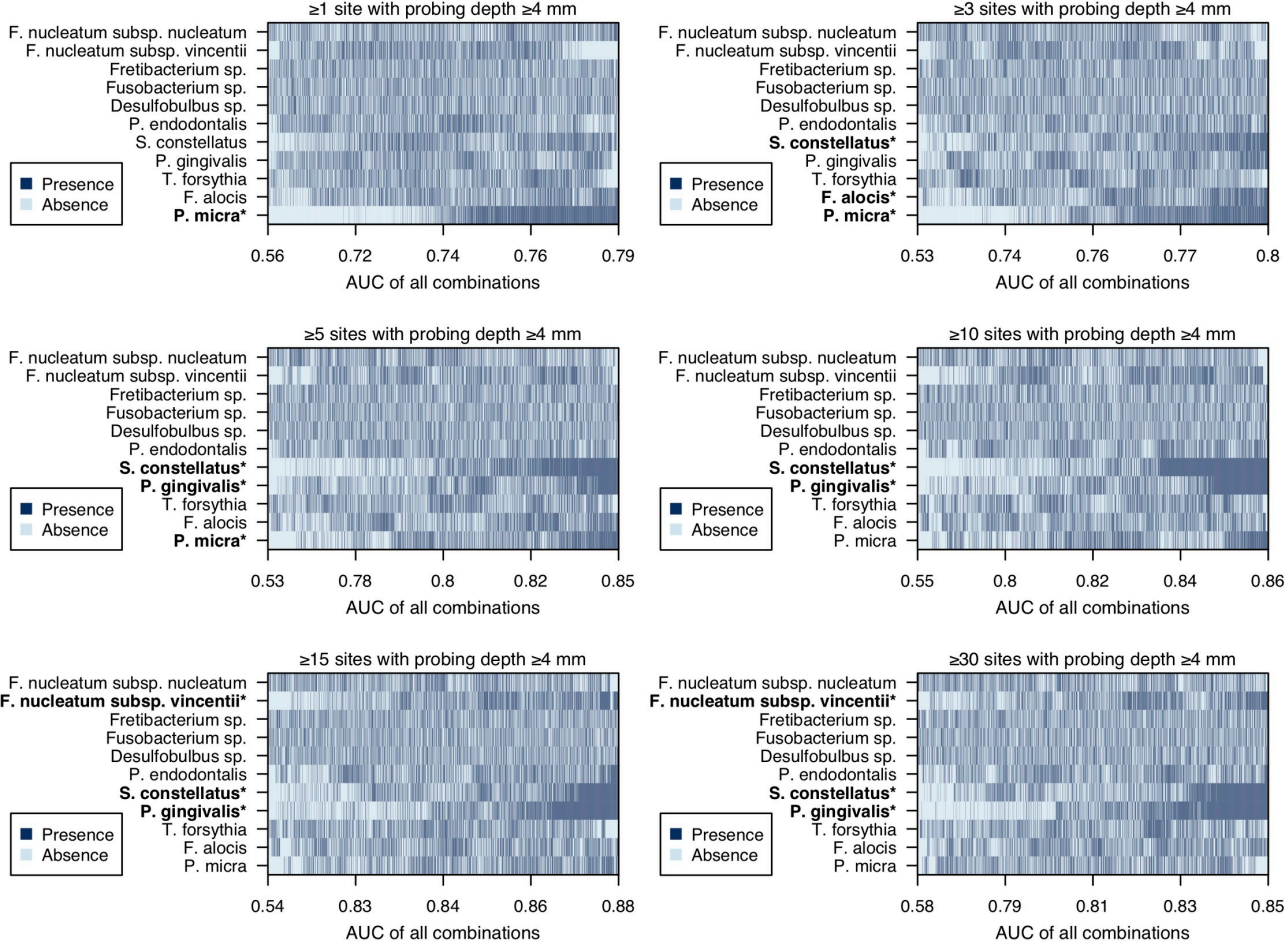
Area under the ROC curves (AUC) values using the total relative abundance of 2,047 combinations of the SUBP bacteria. The column means 2,047 combinations and the all combinations were sorted by their AUC value. Dark blue indicates presence in each combination and light blue indicates absence in each combination. The AUC values of combinations with and without each SUBP bacteria (dark blue vs light blue in each SUBP bacteria) were compared using the Mann-Whitney U test. *P <10^−40^.

To explore bacteria that particularly affect the prediction of periodontal status among 11 SUBP bacteria, we computed 2,047 combinations of 11 SUBP bacteria and performed ROC curve analysis using the total relative abundance of each combination. In Fig 2, all combinations are sorted in descending order of their AUC value from the right in six panels with different criteria for periodontal status, and the presence or absence of component SUBP bacteria in each combination is indicated as dark blue or light blue, respectively. An asterisk at the bacterial name indicates a significant difference between AUC values of combinations with or without SUBP bacteria using the Mann-Whitney U test. The combinations including *Parvimonas micra* demonstrated significantly higher AUC values than combinations without *P*. *micra* in the relatively mild criteria (≥1, ≥3, and ≥5 sites with PD ≥4 mm). In contrast, combinations including *Streptococcus constellatus*, *Porphyromonas gingivalis*, and *Fusobacterium nucleatum* subsp. *vincentii* showed significantly higher AUC values in the severe criteria (≥15 and ≥30 sites with PD ≥4 mm).

## Discussion

The present study validated the clinical utility of SUBP bacteria in the salivary microbiota for periodontitis detection. We found that the total relative abundance distinguished cases of periodontitis with widespread probing sites with a depth of ≥4 mm with a high sensitivity and specificity. When periodontitis was defined as the presence of ≥15 sites with PD ≥4 mm, SUBP bacteria in the saliva detected periodontitis with a sensitivity of 0.90; this result means that this approach could detect periodontitis with few false negatives (actual periodontitis that tested negative). Although there may be false positives with this screening test because of a specificity of 0.70, the AUC value of our screening test for severe periodontitis was 0.87, with a sensitivity of 0.90, which allows the careful selection of subjects with severe periodontitis requiring urgent treatment. Compared to previous approaches that detect periodontitis, such as those using occult blood, enzymes, cytokines, and proteins in saliva, a specificity of 0.70 is not very low [22–26]. The overall performance is considered sufficient for the mass screening of severe periodontitis. Given that the performance was obtained using saliva samples that were independent of the SUBP bacteria identification, the utility of the SUBP bacteria in saliva is credible. In addition, this kind of salivary bacterial test is expected to contribute as a motivator to reassess and improve oral health conditions. Moreover, unlike the time-consuming, invasive, and technical sampling of subgingival plaque, saliva collection is easy and non-invasive, and does not require the expertise of dentists and hygienists. We believe that SUBP bacteria in salivary microbiota have the potential to be used for extensive and non-burdensome screening that estimates the necessity for visiting a dental office to receive periodontal treatment simply by collecting and mailing their own saliva.

The next generation sequencing of the 16S rRNA gene amplicon is one of the most powerful approaches that identifies the bacterial composition in the human oral cavity, and is expected to be a promising clinical microbial test. When the obtained sequences were analyzed, *de novo* clustering approach, which groups all sequences based on sequence identity, is often used [27, 28]. This approach enables us to identify the novel bacterial taxa and estimate ecosystem diversity, independently of existing database. On the other hand, since *de novo* clustering uses all reads of analysis, the clustering result depends on co-analyzed samples. In this study, the reference-based approach was utilized instead of *de novo* clustering method [27, 28]. This approach assigns a candidate bacterial species by reference to the existing database and calculates the relative abundances of SUBP bacteria independently of the co-analyzed samples. Considering the process in the clinical use from the sample collection to the reply of the test results, the analysis independence is necessary. Although the reference-based approach might neglect the existence of bacterial species not listed in the database, it is not significant for the present analysis due to the focus on SUBP bacteria only. We considered this approach indispensable when we analyzed the salivary microbiota collected from clinical test subjects using the next generation sequencer.

We calculated AUC values of all combinations of the 11 SUBP bacteria in the six criteria and found that the SUBP bacteria included in combinations with high AUC values were different for each criterion. In particular, the relative abundance of *P*. *micra* in saliva was effective on mild periodontitis, while that of *S*. *constellatus*, *P*. *gingivalis*, and *F*. *nucleatum* subsp. *vincentii* were effective on severe periodontitis. Interestingly, the prediction performance of *P*. *micra* abundance alone was higher than that of the total abundance of SUBP bacteria in cases with ≥1 site with PD ≥4 mm (S1 Table). Although *P*. *micra* accounted for a higher proportion in saliva of subjects with more ≥4 mm probing sites, similar to other SUBP bacteria, the increase in abundance of *P*. *micra* from healthy to mild cases was greater than that of other SUBP bacteria. These results suggest that *P*. *micra* is increased in saliva immediately after deep periodontal pocket formation, and *S*. *constellatus*, *P*. *gingivalis*, and *F*. *nucleatum* subsp. *vincentii* are increased in saliva after periodontal disease progression. Considering these differences, *P*. *micra* may be involved in the pathogenesis of periodontal disease as an initial subgingival colonizer. Previous studies reported that *P*. *micra* could enhance the growth of *P*. *gingivalis* and gingipain activity and synergistically form biofilms with *F*. *nucleatum* [29, 30]. To test this hypothesis, further studies are warranted that directly examine site-specific subgingival plaque samples collected from periodontal pockets with a wide range of depths.

For sensitivity analysis, we assessed the prediction performance of the test by adding *T*. *denticola* to our evaluation, which is a “red complex” but was not included in SUBP bacteria in our previous study. The best AUC value was obtained for cases in which the periodontal status was the presence of ≥15 sites with PD ≥4 mm, and the results did not significantly change when compared to the result of the analysis that did not include *T*. *denticola* (AUC, 0.87; sensitivity, 0.88; specificity, 0.71).

This study has several potential limitations. First, information on bleeding on probing (BOP) was not obtained in the present study; thus, although there might be a difference in subgingival microbiota and saliva leakage between active periodontitis with BOP and stable periodontitis, it was not feasible to assess the clinical utility of SUBP bacteria considering the activity. Second, saliva samples from the present study were collected from the patient population who visited the dental offices; therefore, further consideration, such as the adjustment of cutoff value, is required to generalize of our findings to the whole population.

In conclusion, the total relative abundance of SUBP bacteria in the salivary microbiota identified patients with severe periodontitis with a high prediction performance. These results suggest that examining SUBP bacteria in saliva may help detect periodontitis patients in mass screening.

## Supporting information

S1 Table Prediction performance of the relative abundance of each SUBP bacteria alone. AUC values (95% CI) are shown. Human oral taxon (HOT) numbers in the human oral microbiome database (HOMD) are given following bacterial names. The best AUC values in each criterion are shown in bold.
(DOCX)
