# Supplementary material for: Clinical utility of subgingival plaque-specific bacteria in salivary microbiota for detecting periodontitis
Source: PLoS One. 2021 Jun 25;16(6):e0253502. doi: 10.1371/journal.pone.0253502 (PMC8232462; doi:10.1371/journal.pone.0253502)
Supplement: S1 Table — AUC values (95% CI) are shown. Human oral taxon (HOT) numbers in the human oral microbiome database (HOMD) are given following bacterial names. The best AUC values in each criterion are shown in bold. (DOCX) [file pone.0253502.s001.docx]

| SUBP bacteria | ≥1 site | ≥3 sites | ≥5 sites | ≥10 sites | ≥15 sites | ≥30 sites |
| --- | --- | --- | --- | --- | --- | --- |
| *Fusobacterium nucleatum* subsp. *nucleatum* HOT698 | 0.57 (0.48-0.66) | 0.57 (0.49-0.65) | 0.60 (0.52-0.68) | 0.61 (0.52-0.70) | 0.64 (0.54-0.73) | 0.62 (0.51-0.74) |
| *Fusobacterium nucleatum* subsp. *vincentii* HOT200 | 0.69 (0.58-0.79) | 0.70 (0.61-0.79) | 0.74 (0.65-0.83) | 0.77 (0.68-0.86) | 0.80 (0.71-0.88) | 0.74 (0.62-0.86) |
| *Fretibacterium* sp. HOT359 | 0.61 (0.55-0.68) | 0.63 (0.56-0.70) | 0.63 (0.56-0.70) | 0.65 (0.56-0.73) | 0.67 (0.58-0.76) | 0.67 (0.56-0.79) |
| *Fusobacterium* sp. HOT370 | 0.56 (0.52-0.59) | 0.53 (0.49-0.58) | 0.53 (0.48-0.58) | 0.55 (0.49-0.60) | 0.54 (0.48-0.60) | 0.58 (0.49-0.66) |
| *Desulfobulbus* sp. HOT041 | 0.64 (0.58-0.69) | 0.60 (0.53-0.67) | 0.62 (0.55-0.69) | 0.62 (0.54-0.71) | 0.64 (0.56-0.73) | 0.64 (0.52-0.75) |
| *Porphyromonas* *endodontalis* HOT273 | 0.66 (0.56-0.75) | 0.66 (0.57-0.75) | 0.68 (0.59-0.77) | 0.72 (0.63-0.82) | 0.75 (0.65-0.84) | 0.73 (0.61-0.86) |
| *Streptococcus* *constellatus* HOT576 | 0.67 (0.57-0.78) | 0.69 (0.60-0.79) | 0.74 (0.66-0.83) | 0.78 (0.69-0.86) | 0.77 (0.69-0.86) | 0.73 (0.62-0.84) |
| *Porphyromonas gingivalis* HOT619 | 0.66 (0.56-0.75) | 0.67 (0.58-0.76) | 0.71 (0.63-0.80) | 0.72 (0.63-0.81) | 0.75 (0.65-0.84) | 0.71 (0.59-0.84) |
| *Tannerella forsythia* HOT613 | 0.62 (0.52-0.73) | 0.62 (0.52-0.71) | 0.65 (0.55-0.74) | 0.66 (0.56-0.76) | 0.69 (0.59-0.79) | 0.70 (0.58-0.81) |
| *Filifactor alocis* HOT539 | 0.63 (0.56-0.70) | 0.68 (0.62-0.75) | 0.69 (0.62-0.76) | 0.70 (0.61-0.78) | 0.73 (0.64-0.82) | 0.80 (0.70-0.91) |
| *Parvimonas micra* HOT111 | **0.78 (0.68-0.87)** | 0.76 (0.67-0.85) | 0.77 (0.68-0.85) | 0.76 (0.67-0.85) | 0.79 (0.71-0.88) | 0.73 (0.61-0.84) |
| Total of 11 SUBP bacteria | 0.76 (0.67-0.85) | **0.78 (0.70-0.87)** | **0.83 (0.76-0.90)** | **0.85 (0.78-0.92)** | **0.87 (0.81-0.93)** | **0.83 (0.76-0.91)** |

AUC values (95% CI) are shown. Human oral taxon (HOT) numbers in the human oral microbiome database (HOMD) are given following bacterial names. The best AUC values in each criterion are shown in bold.
